# Characterization of Extensively Drug-Resistant (XDR) Carbapenemase-Producing *Enterobacterales* (CPE) in Canada from 2019 to 2020

**DOI:** 10.1128/spectrum.00975-22

**Published:** 2022-08-11

**Authors:** Jessica J. Bartoszko, Robyn Mitchell, Kevin Katz, Michael Mulvey, Laura Mataseje

**Affiliations:** a Centre for Communicable Diseases and Infection Control, Public Health Agency of Canada, Ottawa, Ontario, Canada; b Department of Infection Prevention and Control, North York General Hospital, Toronto, Ontario, Canada; c National Microbiology Laboratory, Public Health Agency of Canada, Winnipeg, Manitoba, Canada; Emory University School of Medicine

**Keywords:** AMR, ARO, CPE, antimicrobial resistance, carbapenease-producing *Enterobacterales*, drug-resistant

## Abstract

Data regarding the epidemiology of extensively drug-resistant (XDR) carbapenemase-producing *Enterobacterales* (CPE) in Canada are scarce. Among CPE patients identified by the Canadian Nosocomial Infection Surveillance Program, the following were each significantly associated with XDR status: international travel history; CPE acquisition from a health care exposure abroad; presence of the New Delhi metallo-β-lactamase (NDM) carbapenemase gene; E. coli sequence type (ST) 167, ST405, and ST648; *E. cloaceae* ST177; C. freundii ST22; and resistance to all antimicrobials except colistin, tigecycline, and ceftazidime-avibactam.

**IMPORTANCE** Extensively drug-resistant (XDR) carbapenemase-producing *Enterobacterales* (CPE) are a global public health concern. XDR CPE are among the most drug-resistant and difficult-to-treat bacteria, and infected patients are likely to experience adverse outcomes. Because XDR status further reduces effective therapeutic options, it is critical for clinicians to consider resistance and therapeutic options not only in the context of a patient with CPE but also in the context of potential XDR status. Our study reports on patient characteristics associated with the acquisition of an XDR CPE. Our study also reports on the species and carbapenemases associated with XDR status among *Enterobacterales* identified in Canada. Among a panel of 22 antibiotics, including novel combination drugs, we showed which retained the highest activity against XDR CPE, which may help guide the selection of antibiotic treatments.

## OBSERVATION

Carbapenemase-producing *Enterobacterales* (CPE) are a global public health concern (1, 2). Drug resistance in CPE is well-described and is often associated with multiple different resistance determinants that are harbored on mobile elements (1). Most alarming are the extensively drug-resistant (XDR) CPE, for which antibiotic treatment options are markedly limited (i.e., those susceptible to ≤2 antimicrobial classes), resulting in worse clinical outcomes for patients (1). Data characterizing the epidemiology of XDR CPE in Canada are scarce. Understanding their epidemiology can improve the appropriateness of empirical antibiotic selection, a strong predictor of patient outcomes (1, 3). Further, it can help tailor infection prevention and control (IPC) practices to meet Canadian needs. Thus, we describe the characteristics and the resistance profiles of XDR CPE in Canada.

The Canadian Nosocomial Infection Surveillance Program (CNISP) has conducted surveillance of CPE among adult and pediatric inpatients and outpatients since 2010 (4, 5). Because the antibiotic susceptibility testing of CPE isolates against the newer combination drugs, ceftazidime-avibactam, meropenem-vaborbactam, and imipenem/relebactam, began in 2019, we restricted our surveillance period to 2019 and 2020. Participating acute care facilities submitted isolates that exhibited nonsusceptibility to imipenem, meropenem, or ertapenem to the National Microbiology Laboratory (Winnipeg, Canada) for minimum inhibitory concentration confirmation using imipenem, meropenem, and/or ertapenem Etest strips (bioMérieux, St. Laurent, Quebec, Canada) (6). Broth microdilutions (Sensititre, panel CAN1MSTF) were performed to determine the antibiotic susceptibilities, following the Clinical and Laboratory Standards Institute interpretations (6). Isolates were sequenced using the Illumina NextSeq platform. Carbapenemase (github.com/phac-nml/staramr) and multilocus sequence typing profiles (pubmlst.org) were extracted from whole-genome sequencing data (7). Using the novel Canadian recommendation for the laboratory interpretation of XDR organisms, we defined CPE isolates as XDR if they were resistant to five or six of the following six antibiotic groups: tobramycin or gentamicin, piperacillin-tazobactam, imipenem or meropenem, cefotaxime or ceftriaxone or ceftazidime, ciprofloxacin, or trimethoprim-sulfamethoxazole (8). Through chart review, IPC professionals completed standardized questionnaires for patients harboring laboratory-confirmed CPE, and, using the standardized definitions detailed in our published surveillance protocol, determined the likely source of CPE acquisition (4, 5).

Because a single CPE patient could harbor more than one unique CPE isolate (e.g., differing by organism or carbapenemase gene), we reported demographic and clinical data by the number of unique patients, and we reported molecular and microbiological data by the number of unique isolates. For analyses pertaining to newer combination drugs, we created CPE isolate subsets, each of which was restricted to isolates that harbored carbapenemase genes that the beta-lactamase inhibitor in question could inhibit (9). Using R version 4.1.1, we performed chi-square and Fisher’s exact two-sided tests to compare XDR CPE to non-XDR CPE (10). We set the criterion for statistical significance at α = 0.05.

We identified 482 unique CPE isolates from 440 patients across 33 of 72 participating facilities. Over half of the patients (259/440, 59%) were colonized or infected with an XDR CPE. The incidence rate for XDR CPE significantly decreased from 0.13 per 1,000 admissions in 2019 to 0.10 per 1,000 admissions in 2020 (*P* = 0.048). Table 1 summarizes the demographic data available for the CPE patients, stratified by XDR status.

**TABLE 1:** Summary of available demographics from patients harboring carbapenemase-producing *Enterobacterales* (*N* = 440)

Characteristic	Overall (*N* = 440)^*a*^	Non-XDR (*N* = 181)^*a*^	XDR (*N* = 259)^*a*^	*P* value^*a*^
Age				0.36^*b*^
Median (interquartile range)	66 (52, 76)	66 (54, 77)	66 (51, 75)	
Sex				0.81^*c*^
Female	189/440 (43%)	79/181 (44%)	110/259 (42%)	
Male	251/440 (57%)	102/181 (56%)	149/259 (58%)	
Patient Status				0.47^*c*^
Emergency Room	28/440 (6.4%)	10/181 (5.5%)	18/259 (6.9%)	
Inpatient	389/440 (88%)	159/181 (88%)	230/259 (89%)	
Outpatient	23/440 (5.2%)	12/181 (6.6%)	11/259 (4.2%)	
Infection Site				0.62^*d*^
Blood	24/440 (5.5%)	8/181 (4.4%)	16/259 (6.2%)	
Other	11/440 (2.5%)	3/181 (1.7%)	8/259 (3.1%)	
Sputum/Endotracheal secretions/Bronchoalveolar lavage	11/440 (2.5%)	3/181 (1.7%)	8/259 (3.1%)	
Skin/soft tissue	6/440 (1.4%)	3/181 (1.7%)	3/259 (1.2%)	
Stool/rectal swab	334/440 (76%)	144/181 (80%)	190/259 (73%)	
Surgical site	2/440 (0.5%)	1/181 (0.6%)	1/259 (0.4%)	
Urine	43/440 (9.8%)	14/181 (7.7%)	29/259 (11%)	
Wound	9/440 (2.0%)	5/181 (2.8%)	4/259 (1.5%)	
Source of acquisition				**0.019** ^*c*^
Community-associated	28/337 (8.3%)	12/138 (8.7%)	16/199 (8.0%)	
Healthcare-associated (outside Canada)	66/337 (20%)	17/138 (12%)	49/199 (25%)	
Healthcare-associated (within Canada)	243/337 (72%)	109/138 (79%)	134/199 (67%)	
Geographic Region				**0.033** ^*d*^
Central^*e*^	387/440 (88%)	167/181 (92%)	220/259 (85%)	
East^*f*^	5/440 (1.1%)	0/181 (0%)	5/259 (1.9%)	
West^*g*^	48/440 (11%)	14/181 (7.7%)	34/259 (13%)	
Infections/Colonizations				0.96^*c*^
Colonization	344/406 (85%)	140/165 (85%)	204/241 (85%)	
Infection	62/406 (15%)	25/165 (15%)	37/241 (15%)	

a n/N (%); denominators may vary, as some variables have missing data. Bolded entries are those for which there was a statistically significant association.

b Wilcoxon rank sum test.

c Pearson's chi-square test.

d Fisher's exact test.

e Quebec, Ontario.

f Prince Edward Island, New Brunswick, Nova Scotia, Newfoundland, and Labrador.

g British Columbia, Alberta, Saskatchewan, and Manitoba.

International travel in the year prior to positive culture was significantly more common in XDR patients than in non-XDR patients (80/183, 44% versus 32/122, 26%; *P* = 0.002). Specifically, we observed a significant association between XDR status and travel to South Asia (Nepal, India, Pakistan, Afghanistan, Sri Lanka, or Iran; *P* = 0.010). For those who had data available, over three quarters of the international travelers received medical care while abroad (79/100, 79%). Among patients with health care-associated CPE, acquisition from health care exposure outside Canada compared to within Canada was significantly more common among XDR patients than among non-XDR patients (49/183, 27% versus 17/126, 13%; *P* = 0.005).

Table 2 presents the distribution of genera, carbapenemase genes, and sequence types among CPE isolates, stratified by XDR status. We identified four dominant genera: Escherichia spp. (164/482, 34%), Klebsiella spp. (113/482, 23%), Enterobacter spp. (111/482, 23%), and *Citrobacter* spp. (78/482, 16%). Presence of the Ambler class B carbapenemase *bla*_NDM_ was significantly more common in XDR CPEs than in non-XDR CPEs (130/281, 46% versus 23/201, 11%; *P* < 0.001), whereas the presence of the Ambler class A carbapenemases *bla*_KPC_ and *bla*_NMC_ was significantly less common. Results failed to show a significant association between any other carbapenemase and XDR status. The most common sequence types (present in >10 isolates; ST) were: ST167, ST38, ST405, ST410, and ST648 among E. coli, ST177 among Enterobacter spp., and ST22 among *Citrobacter* spp. The frequency of C. freundii ST22 was likely driven by a hospital outbreak and due to greater diversity among Klebsiella STs, none were classified as common. While results failed to show an association between species and XDR status, when we compared the occurrence of STs, we observed that E. coli ST167, ST405, and ST648, E. cloaceae ST177, and C. freundii ST22 were significantly more common among XDR isolates (Table 2). Specifically, *bla*_NDM_ was the most common carbapenemase among CPE isolates harboring E. coli ST167 (16/18, 89%) and ST405 (15/16, 94%). Further, *bla*_NDM_ was the only carbapenemase among the CPE isolates harboring E. coli ST648 (13/13, 100%) and E. cloaceae ST177 (13/13, 100%).

**TABLE 2:** Summary of available molecular and microbiological characteristics among carbapenemase-producing *Enterobacterales* isolates (*N* = 482)

Characteristic	Overall (*N* = 482)^*a*^	Non-XDR (*N* = 201)^*a*^	XDR (*N* = 281)^*a*^	*P* value^*a*^
GES	1/482 (0.2%)	0/201 (0%)	1/281 (0.4%)	>0.99^*b*^
IMP	2/482 (0.4%)	2/201 (1.0%)	0/281 (0%)	0.17^*b*^
KPC	204/482 (42%)	116/201 (58%)	88/281 (31%)	**<0.001** ^*c*^
NDM	153/482 (32%)	23/201 (11%)	130/281 (46%)	**<0.001** ^*c*^
NDM, OXA-48	17/482 (3.5%)	0/201 (0%)	17/281 (6.0%)	**<0.001** ^*c*^
NMC	11/482 (2.3%)	11/201 (5.5%)	0/281 (0%)	**<0.001** ^*b*^
OXA-48	87/482 (18%)	43/201 (21%)	44/281 (16%)	0.11^*c*^
OXA-48, GES	1/482 (0.2%)	1/201 (0.5%)	0/281 (0%)	0.42^*b*^
SME	3/482 (0.6%)	3/201 (1.5%)	0/281 (0%)	0.072^*b*^
VIM	3/482 (0.6%)	2/201 (1.0%)	1/281 (0.4%)	0.57^*b*^
Enterobacter spp.^*d*^	111/482 (23%)	55/201 (27%)	56/281 (20%)	0.056^*c*^
ST177	13/110 (12%)	0/54 (0%)	13/56 (23%)	**<0.001** ^*c*^
*Citrobacter* spp.^*e*^	78/482 (16%)	30/201 (15%)	48/281 (17%)	0.53^*c*^
ST22	28/66 (42%)	3/22 (14%)	25/44 (57%)	**<0.001** ^*c*^
E. coli	164/482 (34%)	61/201 (30%)	103/281 (37%)	0.15^*c*^
ST167	18/164 (11%)	1/61 (1.6%)	17/103 (17%)	**0.003** ^*c*^
ST38	15/164 (9.1%)	12/61 (20%)	3/103 (2.9%)	**<0.001** ^*c*^
ST405	16/164 (9.8%)	0/61 (0%)	16/103 (16%)	**0.001** ^*c*^
ST410	13/164 (7.9%)	3/61 (4.9%)	10/103 (9.7%)	0.4^*b*^
ST648	13/164 (7.9%)	0/61 (0%)	13/103 (13%)	**0.002** ^*b*^
Klebsiella spp.^*f*^	113/482 (23%)	46/201 (23%)	67/281 (24%)	0.81^*c*^
Other species^*g*^	17/482 (3.5%)	9/201 (4.5%)	8/281 (2.8%)	0.34^*c*^

a n/N (%); denominators may vary, as some variables have missing data and/or proportions are reported for a subset of data. Bolded entries are those for which there was a statistically significant association.

b Fisher's exact test.

c Pearson's chi-square test.

d 88 Enterobacter cloacae and 23 others.

e 51 Citrobacter freundii and 27 others.

f 97 Klebsiella pneumonia, 13 Klebsiella oxytoca, 1 Klebsiella aerogenes, and 2 others.

g *Serratia* spp. (8/482), *Kluyvera* spp. (2/482), *Morganella* spp. (1/482), *Raoultella* spp. (4/482), *Pantoea* spp. (1/482), and Proteus spp. (1/482).

Among the 22 antibiotics included in the susceptibility testing, results failed to show an association between XDR status and resistance for three of them: colistin, tigecycline, and ceftazidime-avibactam (Table 3). For newer combination drugs, compared to non-XDR isolates, we found that XDR isolates exhibited significantly higher percent resistance to meropenem-vaborbactam (5/88, 6% versus 0/130, 0%; *P* = 0.006) and imipenem/relebactam (18/132, 14% versus 7/174, 4%; *P* = 0.006) but not to ceftazidime-avibactam (2/132, 2% versus 2/174, 1%; *P* > 0.9). Overall, percent resistance to novel combination therapies among XDR CPE isolates ranged from 2% for ceftazidime-avibactam to 14% for imipenem/relebactam. Due to the lack of activity of these combination drugs against NDM-producers, we excluded isolates harboring NDM-type carbapenemase genes from the aforementioned three analyses. Data regarding the activity of imipenem/relebactam and ceftazidime-avibactam against OXA-48-type carbapenemases are variable (9). Here, we observed that OXA-48-type carbapenemases were significantly associated with resistance to imipenem/relebactam (resistant [R]: 14/25, 56% versus intermediate [I]: 7/20, 35% versus susceptible [S]: 66/261, 25%; *P* = 0.004), but not to ceftazidime-avibactam (R: 0/4, 0% versus S: 87/302, 29%; *P* = 0.6).

**TABLE 3:** Antibiotic susceptibilities from 2019 to 2020 for all carbapenemase-producing *Enterobacterales* isolates (*N* = 482)

Characteristic^*d*^	Overall (*N* = 482)^*a*^	Non-XDR (*N* = 201)^*a*^	XDR (*N* = 281)^*a*^
Amikacin			
I	16/482 (3.3%)	1/201 (0.5%)	15/281 (5.3%)
R	48/482 (10.0%)	2/201 (1.0%)	46/281 (16%)
S	418/482 (87%)	198/201 (99%)	220/281 (78%)
Aztreonam			
I	20/481 (4.2%)	11/201 (5.5%)	9/280 (3.2%)
R	377/481 (78%)	138/201 (69%)	239/280 (85%)
S	84/481 (17%)	52/201 (26%)	32/280 (11%)
Cefepime			
R	308/481 (64%)	69/201 (34%)	239/280 (85%)
S	74/481 (15%)	66/201 (33%)	8/280 (2.9%)
SDD	99/481 (21%)	66/201 (33%)	33/280 (12%)
Ceftazidime			
I	9/482 (1.9%)	8/201 (4.0%)	1/281 (0.4%)
R	418/482 (87%)	141/201 (70%)	277/281 (99%)
S	55/482 (11%)	52/201 (26%)	3/281 (1.1%)
Ceftolozoane/tazobactam			
I	9/481 (1.9%)	5/201 (2.5%)	4/280 (1.4%)
R	412/481 (86%)	139/201 (69%)	273/280 (98%)
S	60/481 (12%)	57/201 (28%)	3/280 (1.1%)
Ceftriaxone			
I	2/482 (0.4%)	2/201 (1.0%)	0/281 (0%)
R	445/482 (92%)	164/201 (82%)	281/281 (100%)
S	35/482 (7.3%)	35/201 (17%)	0/281 (0%)
Ciprofloxacin			
I	32/482 (6.6%)	28/201 (14%)	4/281 (1.4%)
R	345/482 (72%)	73/201 (36%)	272/281 (97%)
S	105/482 (22%)	100/201 (50%)	5/281 (1.8%)
Doxycycline			
I	62/481 (13%)	21/201 (10%)	41/280 (15%)
R	207/481 (43%)	55/201 (27%)	152/280 (54%)
S	212/481 (44%)	125/201 (62%)	87/280 (31%)
Ertapenem			
I	37/482 (7.7%)	33/201 (16%)	4/281 (1.4%)
R	416/482 (86%)	143/201 (71%)	273/281 (97%)
S	29/482 (6.0%)	25/201 (12%)	4/281 (1.4%)
Gentamicin			
I	17/482 (3.5%)	10/201 (5.0%)	7/281 (2.5%)
R	163/482 (34%)	24/201 (12%)	139/281 (49%)
S	302/482 (63%)	167/201 (83%)	135/281 (48%)
Levofloxacin			
I	45/481 (9.4%)	20/201 (10.0%)	25/280 (8.9%)
R	287/481 (60%)	50/201 (25%)	237/280 (85%)
S	149/481 (31%)	131/201 (65%)	18/280 (6.4%)
Meropenem			
I	46/482 (9.5%)	32/201 (16%)	14/281 (5.0%)
R	326/482 (68%)	88/201 (44%)	238/281 (85%)
S	110/482 (23%)	81/201 (40%)	29/281 (10%)
Minocycline			
I	59/481 (12%)	15/201 (7.5%)	44/280 (16%)
R	138/481 (29%)	34/201 (17%)	104/280 (37%)
S	284/481 (59%)	152/201 (76%)	132/280 (47%)
Piperacillin-tazobactam			
I	26/482 (5.4%)	26/201 (13%)	0/281 (0%)
R	432/482 (90%)	151/201 (75%)	281/281 (100%)
S	24/482 (5.0%)	24/201 (12%)	0/281 (0%)
Tobramycin			
I	64/482 (13%)	34/201 (17%)	30/281 (11%)
R	210/482 (44%)	24/201 (12%)	186/281 (66%)
S	208/482 (43%)	143/201 (71%)	65/281 (23%)
Trimethoprim/sulfamethoxazole			
R	362/482 (75%)	97/201 (48%)	265/281 (94%)
S	120/482 (25%)	104/201 (52%)	16/281 (5.7%)
Ceftazidime-avibactam^*b*^			
R	4/306 (1.3%)	2/174 (1.1%)	2/132 (1.5%)
S	302/306 (99%)	172/174 (99%)	130/132 (98%)
Meropenem-vaborbactam^*c*^			
I	3/218 (1.4%)	1/130 (0.8%)	2/88 (2.3%)
R	5/218 (2.3%)	0/130 (0%)	5/88 (5.7%)
S	210/218 (96%)	129/130 (99%)	81/88 (92%)
Imipenem/relebactam^*b*^			
I	20/306 (6.5%)	14/174 (8.0%)	6/132 (4.5%)
R	25/306 (8.2%)	7/174 (4.0%)	18/132 (14%)
S	261/306 (85%)	153/174 (88%)	108/132 (82%)
Tigecycline			
I	7/482 (1.5%)	2/201 (1.0%)	5/281 (1.8%)
R	1/482 (0.2%)	0/201 (0%)	1/281 (0.4%)
S	474/482 (98%)	199/201 (99%)	275/281 (98%)
Colistin			
I	454/471 (96%)	186/195 (95%)	268/276 (97%)
R	17/471 (3.6%)	9/195 (4.6%)	8/276 (2.9%)
Plazomicin			
I	6/481 (1.2%)	5/201 (2.5%)	1/280 (0.4%)
R	45/481 (9.4%)	2/201 (1.0%)	43/280 (15%)
S	430/481 (89%)	194/201 (97%)	236/280 (84%)

a n/N (%); denominators may vary, as some variables have missing data.

b Isolates carrying NDM-type carbapenemases are excluded.

c Isolates carrying NDM-type and OXA-type carbapenemases are excluded.

d R: resistant, I: intermediate, S: susceptible, SDD: susceptible dose-dependent.

Among the 51 patients infected with CPE for which outcome data were available, 10 deaths were reported (20%). Results failed to show a statistically significant difference in 30-day all-cause in-hospital mortality between XDR and non-XDR patients (5/29, 17% versus 5/22, 23%; *P* = 0.7).

We present novel data regarding the epidemiology of XDR CPE, the most resistant and difficult-to-treat pathogens, in Canada. The travel and medical history risk factors that we identified are consistent with those described in the literature for CPE acquisition in general (11). Our findings align with surveillance from Greece and Spain, which found that the activity of ceftazidime-avibactam was higher than that of imipenem/relebactam against *Enterobacterales* harboring OXA-48-type carbapenemases (12, 13). Further, this report applies novel Canadian recommendations for the laboratory interpretation of XDR clinical isolates to national surveillance data (8). These recommendations are the first of their kind in Canada, and they have been modified from those originally proposed by the European Centre for Disease Prevention and Control and the Centers for Disease Control and Prevention to consider the Canadian context and Canadian stakeholder input (14). Because relevant data were available for 2 years of surveillance and from acute care facilities only, and because a small number of patients were infected (62/406, 15%), the generalizability of our data and our ability to test for a difference in clinical outcome between the XDR and the non-XDR CPE patients were limited.

Given the increasing spread of XDR CPE, it is critical for clinicians to consider potential XDR status due to the fact that they reduce effective therapeutic options (1, 3). Our reporting of both the patient characteristics associated with XDR status and the resistance profiles associated with XDR CPE isolates may prompt further investigations into CPE diagnosis and may help guide IPC practices and empirical antibiotic selection. Ongoing national surveillance of XDR CPE is required to elucidate their epidemiological profiles in Canada.

### Data availability.

The surveillance protocol is available and is cited within the manuscript text. Data-sharing requests will be considered and reviewed by the Public Health Agency of Canada and by individual site investigators. The BioProject number under which the generated sequencing data can be found is PRJNA855907.
